# Long‐Term Impact of COVID‐19 on Disorders of Gut–Brain Interaction: Incidence, Symptom Burden, and Psychological Comorbidities

**DOI:** 10.1002/ueg2.70005

**Published:** 2025-03-21

**Authors:** Giovanni Marasco, Keren Hod, Luigi Colecchia, Cesare Cremon, Maria Raffaella Barbaro, Giulia Cacciari, Francesca Falangone, Anna Kagramanova, Dmitry Bordin, Vasile Drug, Egidia Miftode, Pietro Fusaroli, Salem Youssef Mohamed, Chiara Ricci, Massimo Bellini, M. Masudur Rahman, Luigi Melcarne, Javier Santos, Beatriz Lobo, Serhat Bor, Suna Yapali, Deniz Akyol, Ferdane Pirincci Sapmaz, Yonca Yilmaz Urun, Tugce Eskazan, Altay Celebi, Huseyin Kacmaz, Berat Ebik, Hatice Cilem Binicier, Mehmet Sait Bugdayci, Munkhtsetseg Banzragch Yağcı, Husnu Pullukcu, Berrin Yalınbas Kaya, Ali Tureyen, İbrahim Hatemi, Elif Sitre Koc, Goktug Sirin, Ali Riza Calıskan, Goksel Bengi, Esra Ergun Alıs, Snezana Lukic, Meri Trajkovska, Dan Dumitrascu, Antonello Pietrangelo, Elena Corradini, Magnus Simren, Jessica Sjolund, Navkiran Tornkvist, Uday C. Ghoshal, Olga Kolokolnikova, Antonio Colecchia, Jordi Serra, Giovanni Maconi, Roberto De Giorgio, Silvio Danese, Piero Portincasa, Antonio Di Sabatino, Marcello Maggio, Elena Philippou, Yeong Yeh Lee, Daniele Salvi, Alessandro Venturi, Claudio Borghi, Marco Zoli, Paolo Gionchetti, Pierluigi Viale, Vincenzo Stanghellini, Giovanni Barbara, GI‐COVID19 study groups

**Affiliations:** ^1^ Department of Medical and Surgical Sciences University of Bologna Bologna Italy; ^2^ IRCCS Azienda Ospedaliero‐Universitaria di Bologna Bologna Italy; ^3^ Department of Nutrition Sciences School of Health Sciences Ariel University Ariel Israel; ^4^ Assuta Medical Centers Tel Aviv Israel; ^5^ Medical‐Surgical Department of Clinical Sciences and Translational Medicine University Sapienza Rome Italy; ^6^ A. S. Loginov Moscow Clinical Scientific Center Moscow Russia; ^7^ Research Institute of Health Organization and Medical Management Moscow Russia; ^8^ Tver State Medical University Tver Russia; ^9^ Russian University of Medicine Moscow Russia; ^10^ Department of Gastroenterology “Grigore T. Popa” University of Medicine and Pharmacy Iasi Romania; ^11^ Department of Infectious Diseases “Grigore T. Popa” University of Medicine and Pharmacy Iasi Romania; ^12^ Gastroenterology Unit Imola Hospital Imola Italy; ^13^ Gastroenterology and Hepatology Unit Internal Medicine Department Faculty of Medicine Zagazig University Zagazig Egypt; ^14^ Department of Experimental and Clinical Sciences University of Brescia Brescia Italy; ^15^ Gastroenterology Unit University of Pisa Pisa Italy; ^16^ Sheikh Russel National Gastroliver Institute and Hospital Dhaka Bangladesh; ^17^ Sabadell ‐ CIBEREHD Centro de Investigación Biomédica en Red Hospital Universitari Parc Taulí Barcelona Spain; ^18^ Gastroenterology Department Vall d'Hebron Hospital Universitari Vall d'Hebron Hospital Campus Barcelona Spain; ^19^ Digestive Physiology and Physiopathology Research Group Vall d'Hebron Research Institute (VHIR) Barcelona Spain; ^20^ Centro de Investigación Biomédica en Red Enfermedades Hepáticas y Digestivas (CIBERhed) Instituto de Salud Carlos III Madrid Spain; ^21^ Ege University Division of Gastroenterology Izmir Turkey; ^22^ Division of Gastroenterology Acibadem University Altunizade Acibadem Hospital Istanbul Turkey; ^23^ Ege University Department of Infectious Diseases Izmir Turkey; ^24^ Division of Gastroenterology University of Health Sciences Keciören Education and Research Hospital Keciören Turkey; ^25^ Division of Gastroenterology Eskisehir City Hospital Eskisehir Turkey; ^26^ Cerrahpasa Faculty of Medicine Division of Gastroenterology Istanbul University‐Cerrahpasa Istanbul Turkey; ^27^ Division of Gastroenterology Kocaeli University Kocaeli Turkey; ^28^ Division of Gastroenterology Adiyaman Education and Research Hospital Adiyaman Turkey; ^29^ Division of Gastroenterology University of Health Sciences Diyabakır Gazi Yasargil Education and Research Hospital Diyarbakır Turkey; ^30^ Division of Gastroenterology Dokuz Eylül University Izmir Turkey; ^31^ Division of Gastroenterology İstanbul Aydın University Florya Liv Hospital Istanbul Turkey; ^32^ Division of Gastroenterology Darıca Farabi Education and Research Hospital Kocaeli Turkey; ^33^ Department of Infectious Diseases İstanbul Aydın University Florya Liv Hospital Istanbul Turkey; ^34^ Clinic for Gastroenterohepatology University Clinical Centre of Serbia Belgrade Serbia; ^35^ Clinic of Gastroenterohepatology Skopje North Macedonia; ^36^ Iuliu Hatieganu University of Medicine and Pharmacy Cluj‐Napoca Romania; ^37^ Internal Medicine Unit Modena University Hospital University of Modena and Reggio Emilia Modena Italy; ^38^ Sahlgrenska University Hospital Gothenburg Sweden; ^39^ Institute of Gastrosciences and Liver Transplantation Apollo Multispeciality Hospitals Kolkata India; ^40^ Medsi Clinical Hospital Otradnoye Russia; ^41^ Gastroenterology Unit Verona University Hospital Verona Italy; ^42^ CIBERehd University Hospital Germans Trias i Pujol Barcelona Spain; ^43^ Gastroenterology Unit Department of Biomedical and Clinical Sciences L.Sacco University Hospital University of Milan Milan Italy; ^44^ Department of Translational Medicine University of Ferrara Ferrara Italy; ^45^ Gastroenterology and Endoscopy IRCCS Ospedale San Raffaele and University Vita‐Salute San Raffaele Milano Italy; ^46^ Division of Internal Medicine “A. Murri” Department of Precision and Regenerative Medicine and Ionian Area, (DiMePre‐J) University of Bari “Aldo Moro” Bari Italy; ^47^ First Department of Internal Medicine Fondazione IRCCS Policlinico San Matteo University of Pavia Pavia Italy; ^48^ Geriatric Clinic Unit Medical Geriatric Rehabilitative Department University Hospital of Parma Parma Italy; ^49^ Department of Life Sciences School of Life and Health Sciences University of Nicosia Nicosia Cyprus; ^50^ School of Medical Sciences Universiti Sains Malaysia Kota Bharu Malaysia

**Keywords:** anxiety, COVID‐19, depression, disorders of gut‐brain interaction, functional dyspepsia, gastrointestinal symptoms, irritable bowel syndrome, post‐infection gastrointestinal disorders

## Abstract

**Background:**

The severe acute respiratory syndrome coronavirus 2 (SARS‐CoV‐2) pandemic has highlighted the potential exacerbation of gastrointestinal symptoms in patients with disorders of gut‐brain interaction (DGBIs). However, the distinct symptom trajectories and psychological burden in patients with post‐COVID‐19 DGBIs compared with patients with pre‐existing irritable bowel syndrome (IBS)/functional dyspepsia (FD) and non‐DGBI controls remain poorly understood.

**Objectives:**

To examine the long‐term gastrointestinal symptom progression and psychological comorbidities in patients with post‐COVID‐19 DGBI, patients with pre‐existing IBS/FD and non‐DGBI controls.

**Methods:**

This post hoc analysis of a prospective multicenter cohort study reviewed patient charts for demographic data and medical history. Participants completed the Gastrointestinal Symptom Rating Scale at four time points: baseline, 1, 6, and 12 months, and the Hospital Anxiety and Depression Scale at 6 and 12 months. The cohort was divided into three groups: (1) post‐COVID‐19 DGBIs (2) non‐DGBI, and (3) pre‐existing IBS/FD, with the post‐COVID‐19 DGBIs group compared to the latter two control groups.

**Results:**

Among 599 eligible patients, 27 (4.5%) were identified as post‐COVID‐19 DGBI. This group experienced worsening abdominal pain, hunger pain, heartburn, and acid regurgitation, unlike symptom improvement or stability in non‐DGBI controls (*p* < 0.001 for all symptoms, except hunger pain, *p* = 0.001). While patients with pre‐existing IBS/FD improved in most gastrointestinal symptoms but worsened in constipation and incomplete evacuation, patients with post‐COVID‐19 DGBI exhibited consistent symptom deterioration across multiple gastrointestinal domains. Anxiety and depression remained unchanged in patients with post‐COVID‐19 DGBI, contrasting with significant reductions in controls (non‐DGBI: *p* = 0.003 and *p* = 0.057; pre‐existing IBS/FD: *p* = 0.019 and *p* = 0.007, respectively).

**Conclusions:**

COVID‐19 infection is associated with the development of newly diagnosed DGBIs and distinct symptom trajectories when compared with patients with pre‐existing IBS/FD. Patients with post‐COVID‐19 DGBI experience progressive gastrointestinal symptom deterioration and persistent psychological distress, underscoring the need for tailored management strategies for this unique subgroup.

## Graphical abstract

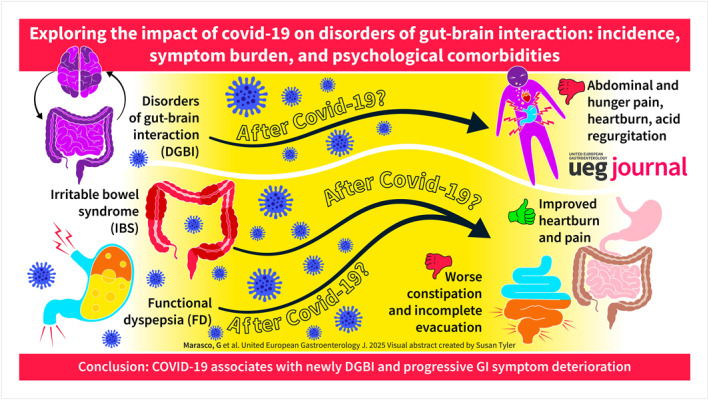

1

**Summary**

- Summarize the established knowledge on this subject
  ◦ Acute infections, including COVID‐19, are associated with an increased risk of developing disorders of gut‐brain interaction (DGBIs) and long‐lasting gastrointestinal (GI) symptoms.
  ◦ The progression of GI symptoms and psychological distress in patients with newly diagnosed DGBI following COVID‐19 remains poorly understood compared to patients with pre‐existing irritable bowel syndrome (IBS)/functional dyspepsia (FD) and healthy controls.
- What are the significant and/or new findings of this study?
  ◦ Patients who developed post‐COVID‐19 DGBI showed an increased prevalence of GI symptoms over time and experienced progressive worsening of abdominal pain, hunger pain, heartburn, and acid regurgitation.
  ◦ Patients with pre‐existing IBS/FD had the highest prevalence of clinically significant symptoms, which improved over time with the exception of constipation, hard stool and incomplete evacuation.
  ◦ Patients with post‐COVID‐19 DGBI exhibited persistent psychological distress, with no improvement in anxiety or depression over time, in contrast to the amelioration observed in both pre‐existing IBS/FD and non‐DGBI controls.

## 2 Introduction

Coronavirus Disease 2019 (COVID‐19), caused by the severe acute respiratory syndrome coronavirus 2 (SARS‐CoV‐2), primarily leads to mild‐to‐moderate respiratory symptoms, although elderly subjects and those with underlying conditions are at higher risk for severe illness [1, 2]. The COVID‐19 pandemic’s global impact has been mitigated by SARS‐CoV‐2 vaccinations, with over 56% of the population fully immunized [3]. Common symptoms of acute infection include cough, fever, and respiratory distress. Research also highlights COVID‐19’s significant gastrointestinal (GI) impact [4, 5], with symptoms such as vomiting, diarrhea, appetite loss, abdominal pain and increased GI bleeding risk [3, 4]. Additionally, over 10% of COVID‐19 cases result in long COVID [5], manifesting in more than 200 symptoms across organ systems, including fatigue, cognitive impairment, and diminished well‐being [6]. Our previous research, found that COVID‐19 survivors had increased susceptibility to develop irritable bowel syndrome (IBS) and long‐term GI symptoms [7]. IBS prevalence was 3.2% among COVID‐19 survivors, compared with 0.5% in non‐COVID‐19 individuals [7]. A meta‐analysis of 10 studies involving 2763 patients confirmed these findings, reporting a pooled odds ratio (OR) of 6.27 for IBS occurrence in patients with COVID‐19 infection versus controls [8].

Acute events often alter chronic disease trajectories, as seen with chronic obstructive pulmonary disease (COPD) exacerbations after respiratory infections or liver failure following systemic infections [9]. Less clear associations can instead be found between GI pathogens and inflammatory bowel disease (IBD) flares [10], even though possible causative mechanisms may be related to alterations of the host's gut microbiota.

Preliminary data suggest COVID‐19 may exacerbate symptoms in patients with disorders of gut‐brain interaction (DGBI), though prior studies had limited sample sizes and design [11, 12].

This study aims to understand whether the trajectory of GI symptoms and psychological distress in patients with post‐COVID‐19 DGBIs differs from that of patients with pre‐existing IBS/FD and/or non‐DGBI controls. By comparing these patients, this may allow for a deeper understanding of the distinct impacts of COVID‐19 on GI health, the progression of psychological comorbidities, and the overall burden of DGBI, helping to inform targeted management strategies and improve long‐term patient outcomes.

## 3 Methods

### 3.1 Study Design and Population

This post hoc analysis utilized data from a multicenter observational cohort study of patients with and without COVID‐19 infection, hospitalized from May to October 2020 across 36 centers in 14 countries. The study design and main findings are published elsewhere [7, 13]. Patients were prospectively enrolled upon hospital admission and followed at 1, 6, and 12 months post‐discharge. Eligible patients were aged 18–85 years with COVID‐19 confirmed by World Health Organization (WHO) criteria, and symptoms requiring hospitalization. The study was approved by IRCCS Policlinico S. Orsola Ethical Committee (April 24th 2020, 399/2020/Oss/AOUBo), written informed consent was required from each patient included in the study, and the study protocol conformed to the ethical guidelines of the 1975 Declaration of Helsinki.

This analysis excluded patients hospitalized for non‐COVID‐19 reasons and with GI conditions other than IBS or FD at baseline. Exclusions included diverticular disease, gastroesophageal reflux disease (GERD), gallstones, chronic liver disease, celiac disease, IBD, *Clostridioides difficile* infection, peptic ulcer, active *Helicobacter pylori* infection, or GI malignancies. Patients with prior GI surgeries were also excluded. The exclusion relied on the clinical diagnoses recorded in patients’ medical files.

All patients with confirmed COVID‐19 infection and no prior history of GI disorders, surgeries, or symptoms, apart from minor symptoms like borborygmus, eructation and increased flatus, were monitored over a 1‐year follow‐up to evaluate the incidence of newly diagnosed DGBI. This study population was divided into three main groups [1]: patients with post‐COVID‐19 DGBIs who met criteria for DGBIs during follow‐up based on Rome IV criteria, including epigastric pain syndrome (EPS), postprandial distress syndrome (PDS), FD, chronic nausea and vomiting, cyclic vomiting syndrome, functional diarrhea (FDr), and IBS, at 6 and/or 12 months [2]; non‐DGBI control patients who did not meet DGBI criteria at any point during the follow‐up period [3]; patients with a pre‐existing diagnosis of IBS or FD who tested positive for COVID‐19. All diagnoses were documented in patients' medical records and established prior to the study through evaluations conducted by professional gastroenterologists at tertiary medical centers in accordance with the Rome IV criteria.

### 3.2 Outcome Measures

Baseline demographic and clinical data were extracted from patients’ medical records, including age, sex, body mass index (BMI), smoking status, alcohol use, physical activity habits, co‐morbidities and chronic medication intake with possible GI effects.

Upon admission (baseline), SARS‐CoV infection was confirmed through laboratory tests, and all patients completed the GI Symptom Rating Scale (GSRS) [14] to document the presence of pre‐COVID‐19 GI symptoms using yes/no questions. The GSRS was subsequently administered at 1‐, 6‐, and 12‐months post‐discharge to evaluate the presence and severity of 15 GI symptoms including abdominal pain, hunger pain, nausea, heartburn, acid regurgitation, diarrhea, loose stools, urgent need for defecation, abdominal distention, constipation, hard stools, feeling of incomplete evacuation, eructation, increased flatus and borborygmus, rated on a 1–7 severity scale. GSRS Scores were analyzed both as continuous variables (severity) and as dichotomous variables using two distinct cut‐off points. The first cut‐off distinguished “no discomfort at all” from all other levels (“very mild,” “mild,” “moderate,” “moderate‐severe,” “severe,” and “very severe”) to assess the presence or absence of GI symptoms. The second cut‐off grouped “no discomfort at all,” “very mild,” and “mild” together versus “moderate,” “moderate‐severe,” “severe,” and “very severe” to evaluate the proportion of patients with clinically significant symptoms. The Hospital Anxiety and Depression Scale (HADS) [15] was used to evaluate depression and anxiety at 6 and 12 months.

### 3.3 Data Analysis

Descriptive analyses of patient demographics, anamnestic characteristics, and psychological disorders were presented as median (interquartile range, IQR) and mean (standard deviation, SD) depending on the distribution. Comparisons between groups for demographic and anamnestic variables, comorbidities, and psychological scores were conducted using the Chi‐square test and Mann‐whitney tests. The Wilcoxon test compared psychological scores within each group from 6 to 12 months.

Crude odds ratios (ORs) for each of the 15 GI symptoms were calculated using logistic regression models. Longitudinal data at baseline, 1, 6, and 12 months were analyzed with generalized estimating equations (GEE) and a linear mixed model. GEE assessed the presence of GSRS symptoms as dichotomous outcomes throughout the study follow‐up, with ORs calculated as pooled ORs for the entire follow‐up period. The linear mixed model assessed the severity of GI symptoms throughout the follow‐up period, and interactions between groups over time. Both models were adjusted for time (months), gender, and psychological factors at 6 and 12 months.

All analyses employed a two‐tailed significance threshold of *p* < 0.05 and false detection rate (FDR) < 0.1. Data analyses were performed using the SPSS statistical package (Version 29, SSPS Inc., Chicago, IL).

## 4 Results

### 4.1 Study Population

**FIGURE 1:**
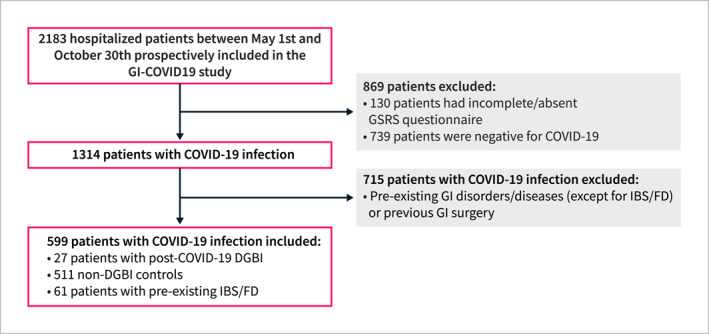
Flowchart of the selection of patients enrolled in the study.

A total of 599 participants with COVID‐19 infection were included in the analysis, comprising 27 (4.5%) without prior GI diseases or surgeries who were identified as post‐COVID‐19 DGBIs, 511 (85.0%) non‐DGBI control participants, and 61 (10.2%) with pre‐existing IBS or FD (only two of whom were diagnosed with both disorders) (Table 1). Among patients with post‐COVID‐19 DGBIs, FD was the most prevalent DGBI (63.0%) consisting of PDS (37.0%) and EPS (29.6%), with one patient diagnosed with both subtypes. Irritable bowel syndrome was reported by 37.0%, chronic nausea vomiting syndrome by 11.1%, cyclic vomiting syndrome and functional diarrhea by 3.7% each (Figure S1). A total of 1584 patients were excluded: 130 due to incomplete or missing GSRS questionnaires, 739 due to negative COVID‐19 testing upon hospital admission, and 715 due to pre‐existing GI diagnoses, previous GI surgeries, or GI symptoms reported prior to COVID‐19 infection (Figure 1).

**TABLE 1:** Demographics and anamnestic information of the study groups.

Demographics and anamnestic characteristics	Post‐COVID‐19 DGBIs (*n* = 27), % (*n*) or median (IQR)	Non‐DGBI (*n* = 511), % (*n*) or median (IQR)	Pre‐existing IBS/FD (*n* = 61), % (*n*) or median (IQR)	*p* value^a^ ^,^ ^b^	FDR^a^ ^,^ ^b^
Age (years)	56.0 (39.0–64.0)	51.0 (36.0–62.0)	49.0 (41.5–59.0)	0.278, 0.469	0.464, > 0.999
Gender, male	69.2 (18)	60.8 (309)	45.0 (27)	0.536, 0.059	0.617, 0.452
BMI (kg/m^2^)	26.3 (24.4–31.4)	26.7 (24.1–30.1)	27.5 (25.5–32.4)	0.485, 0.480	0.604, > 0.999
Smoker status				0.102, 0.219	0.234, > 0.999
No	63.0 (17)	70.9 (358)	78.3 (47)		
Current	3.7 (1)	10.9 (55)	5.0 (3)		
Former	33.3 (9)	18.2 (92)	16.7 (10)		
Regular alcohol consumers	33.3 (9)	14.7 (74)	13.1 (8)	0.024, 0.040	0.118, 0.460
Physical activity (at least 30 min 3 times/week)	40.0 (10)	28.6 (139)	16.1 (9)	0.259, 0.025	0.464, 0.575
Comorbidities					
Neurological	3.7 (1)	2.2 (11)	3.3 (2)	0.464, > 0.999	0.604, > 0.999
Cardiovascular	37.0 (10)	27.4 (140)	32.8 (20)	0.277, 0.888	0.464, > 0.999
Respiratory	3.7 (1)	6.8 (35)	13.1 (8)	> 0.999, 0.265	> 0.999, > 0.999
Hepatological	3.7 (1)	1.4 (7)	1.6 (1)	0.339, 0.522	0.604, > 0.999
Nephrological	3.7 (1)	4.1 (21)	4.9 (3)	> 0.999, > 0.999	> 0.999, > 0.999
Diabetes mellitus	25.9 (7)	14.3 (73)	18.0 (11)	0.101, 0.404	0.234, > 0.999
Metabolic diseases other than diabetes	14.8 (4)	7.0 (36)	18.0 (11)	0.132, > 0.999	0.292, > 0.999
Musculoskeletal	3.7 (1)	2.3 (12)	8.2 (5)	0.492, 0.662	0.604, > 0.999
Psychiatric	0.0 (0)	1.2 (6)	4.9 (3)	> 0.999, 0.550	> 0.999, > 0.999
Gynecological	0.0 (0)	0.2 (1)	1.6 (1)	> 0.999, > 0.999	> 0.999, > 0.999
Urological	0.0 (0)	3.5 (18)	3.3 (2)	> 0.999, > 0.999	> 0.999, > 0.999
Rheumatological	7.4 (2)	2.0 (10)	4.9 (3)	0.117, 0.640	0.246, > 0.999
Allergies	7.4 (2)	2.2 (11)	3.3 (2)	0.135, 0.583	0.292, > 0.999
Autoimmune	7.4 (2)	2.5 (13)	3.3 (2)	0.170, 0.583	0.331, > 0.999
Neoplastic	0.0 (0)	1.6 (8)	4.9 (3)	> 0.999, 0.550	> 0.999, > 0.999
Psychological	3.7 (1)	1.4 (7)	1.6 (1)	0.339, 0.522	0.604, > 0.999
Hematological	0.0 (0)	1.0 (5)	1.6 (1)	> 0.999, > 0.999	> 0.999, > 0.999

*Note*: Post‐COVID‐19 DGBIs group consists of patients with COVID‐19 inflammation who met criteria for DGBIs during follow‐up based on Rome IV criteria. Non‐DGBI group consists of patients with COVID‐19 inflammation who did not meet DGBI criteria at any point during the follow‐up period. The pre‐existing IBS/FD group consists of patients with COVID‐19 inflammation and a baseline diagnosis of IBS or FD based on Rome IV criteria.

Abbreviations: BMI, body mass index; DGBI, disorders of gut‐brain interaction; FD, functional dyspepsia; FDR, false discovery rate; IBS, irritable bowel syndrome; IQR, interquartile range; *n*, number.

^a^ *p*‐values for comparisons between post‐COVID‐19 DGBIs and non‐DGBI controls.

^b^ *p*‐values for comparisons between post‐COVID‐19 DGBIs and patients with pre‐existing IBS/FD.

Baseline characteristics of the overall study population are summarized in Table 1. The majority of participants were male (59.1%, *n* = 354), non‐smokers (70.5%, *n* = 422), physically inactive (less than 30 min 3 times per week, 68.3%, *n* = 409), and infrequent alcohol consumers (84.6%, *n* = 501). The median age was 51.0 years (IQR 36.0–62.0) and the median BMI was 26.8 kg/m^2^ (IQR 24.1–30.8). No significant differences were observed between the groups for most demographic and medical history variables (Table 1).

### 4.2 Depression and Anxiety Score

Anxiety and depression levels were significantly higher at both 6 and 12 months among patients with post‐COVID‐19 DGBIs (anxiety at 6 months: 7.1 ± 5.4; anxiety at 12 months: 6.0 ± 4.6; depression at 6 months: 6.3 ± 5.3; depression at 12 months: 5.5 ± 4.9) compared to non‐DGBI controls (anxiety at 6 months: 3.3 ± 3.4, *p* < 0.001, FDR = 0.001; anxiety at 12 months: 3.1 ± 3.5, *p* = 0.001, FDR = 0.002; depression at 6 months: 3.4 ± 3.9, *p* = 0.004, FDR = 0.004; depression at 12 months: 2.9 ± 3.6, *p* = 0.003, FDR = 0.004). In contrast, no significant differences in anxiety or depression levels were observed between patients with post‐COVID‐19 DGBIs and pre‐existing IBS/FD at either 6 or 12 months. Anxiety and depression levels of post‐COVID‐19 DGBI patients remained unchanged over time in contrast to both pre‐existing IBS/FD and non‐DGBI groups, which demonstrated significant decreases in anxiety (*p* = 0.019, FDR = 0.019; *p* = 0.003, FDR = 0.006, respectively) and depression (*p* = 0.007, FDR = 0.014; *p* = 0.057, FDR = 0.057, respectively) over the same period (Table 2).

**TABLE 2:** Depression and anxiety scores at 6 months compared to those at 12 months across the study groups.

	Post‐COVID‐19 DGBIs (*n* = 27)				Non‐DGBI controls (*n* = 511)				Pre‐existing IBS/FD (*n* = 61)			
	6 months	12 months	*p*‐value	FDR	6 months	12 months	*p*‐value	FDR	6 months	12 months	*p*‐value	FDR
Depression score	6.26 ± 5.33	5.50 ± 4.99	0.085^a^	0.170	3.39 ± 3.86	2.86 ± 3.57	0.057	0.057	6.17 ± 4.45	3.17 ± 4.14	0.007^a^	0.014
Anxiety score	7.15 ± 5.40	6.04 ± 4.63	0.100^a^	0.100	3.34 ± 3.45	3.05 ± 3.48	0.003	0.006	5.94 ± 4.32	4.10 ± 4.58	0.019^a^	0.019

*Note*: All results are presented as mean ± SD. Depression and anxiety were evaluated by the HADS (hospital anxiety and depression scale). Post‐COVID‐19 DGBIs group consists of patients with COVID‐19 inflammation who met criteria for DGBIs during follow‐up based on Rome IV criteria. Non‐DGBI group consists of patients with COVID‐19 inflammation who did not meet DGBI criteria at any point during the follow‐up period. The pre‐existing IBS/FD group consists of patients with COVID‐19 inflammation and a baseline diagnosis of IBS or FD based on Rome IV criteria.

Abbreviations: DGBI, disorders of gut‐brain interaction; FD, functional dyspepsia; FDR, false discovery rate; IBS, irritable bowel syndrome; SD, standard deviation.

^a^ Wilcoxon test.

### 4.3 GI Symptoms

#### 4.3.1 Prevalence of Post‐Infection (PI) Symptoms

At baseline, patients with post‐COVID‐19 DGBIs showed a notable prevalence of diarrhea (59.3%), loose stool (48.1%), urgency (34.6%), and feeling of incomplete evacuation (22.2%) compared with non‐DGBI controls. Most abdominal and upper GI symptoms were significantly less frequent in patients with post‐COVID‐19 DGBIs compared with the pre‐existing IBS/FD group at baseline, particularly abdominal distention (14.8% vs. 54.1%, *p* < 0.001, FDR = 0.040) (Table 3).

**TABLE 3:** Presence of GI symptoms and clinically significant GI symptoms in the study groups.

	Presence of GI symptoms^a^					Clinically significant GI symptoms^b^				
	Post‐COVID‐19 DGBIs (*n* = 27)	Non‐DGBI (*n* = 511)	Pre‐existing IBS/FD (*n* = 61)	*p*‐value^c^ ^,^ ^d^	FDR^c^ ^,^ ^d^	Post‐COVID‐19 DGBIs (*n* = 27)	Non‐DGBI (*n* = 511)	Pre‐existing IBS/FD (*n* = 61)	*p*‐value^c^ ^,^ ^d^	FDR^c^ ^,^ ^d^
Abdominal symptoms										
Abdominal pain										
Baseline	26.9 (7)	21.5 (109)	50.0 (30)	0.474, 0.059	0.580, 0.322	7.7 (2)	12.1 (61)	35.0 (21)	0.756, 0.008	0.840, 0.480
1 month of follow‐up	23.1 (6)	8.2 (38)	30.9 (17)	0.022, 0.600	0.057, 0.947	11.5 (3)	2.1 (10)	18.2 (10)	0.025, 0.533	0.083, 0.842
6 months of follow‐up	44.4 (12)	9.3 (40)	40.0 (18)	< 0.001, 0.807	0.040, > 0.999	18.5 (5)	2.3 (10)	20.0 (9)	< 0.001, > 0.999	0.010, > 0.999
12 months of follow‐up	50.0 (13)	8.1 (25)	35.7 (15)	< 0.001, 0.313	0.030, 0.854	15.4 (4)	2.9 (9)	11.9 (5)	0.013, 0.723	0.052, 0.964
Abdominal distention										
Baseline	14.8 (4)	12.4 (63)	54.1 (33)	0.763, < 0.001	0.832, 0.040	11.1 (3)	6.5 (33)	37.7 (23)	0.415, 0.012	0.638, 0.360
1 month of follow‐up	23.1 (6)	10.0 (46)	33.3 (19)	0.047, 0.443	0.094, 0.917	7.7 (2)	2.7 (13)	17.5 (10)	0.180, 0.324	0.338, 0.778
6 months of follow‐up	44.4 (12)	15.6 (67)	46.7 (21)	< 0.001, > 0.999	0.020, > 0.999	11.1 (3)	4.9 (21)	26.7 (12)	0.163, 0.143	0.315, 0.660
12 months of follow‐up	34.6 (9)	16.8 (52)	47.6 (20)	0.033, 0.324	0.073, 0.845	11.5 (3)	7.1 (22)	21.4 (9)	0.427,0.348	0.625, 0.803
Borborygmus										
Baseline	14.8 (4)	16.7 (85)	41.0 (25)	> 0.999, 0.026	> 0.999, 0.195	11.1 (3)	8.3 (42)	21.3 (13)	0.488, 0.371	0.637, 0.824
1 month of follow‐up	15.4 (4)	8.5 (39)	22.8 (13)	0.273, 0.563	0.372, 0.994	0.0 (0)	1.5 (7)	14.0 (8)	> 0.999, 0.052	> 0.999, 0.446
6 months of follow‐up	29.6 (8)	14.4 (62)	34.8 (16)	0.049, 0.798	0.095, > 0.999	11.1 (3)	4.4 (19)	19.6 (9)	0.133, 0.516	0.333, 0.885
12 months of follow‐up	19.2 (5)	13.9 (43)	31.0 (13)	0.396, 0.399	0.495, 0.887	11.5 (3)	4.5 (14)	19.0 (8)	0.136, 0.512	0.302, 0.904
Increased flatus										
Baseline	22.2 (6)	12.0 (61)	32.8 (20)	0.131, 0.448	0.212, 0.867	11.1 (3)	7.8 (40)	16.4 (10)	0.468, 0.747	0.638, 0.896
1 month of follow‐up	19.2 (5)	10.0 (46)	26.3 (15)	0.176, 0.587	0.271, 0.952	3.8 (1)	3.0 (14)	10.5 (6)	0.558, 0.425	0.712, 0.911
6 months of follow‐up	25.9 (7)	15.4 (66)	43.5 (20)	0.172, 0.209	0.272, 0.738	7.4 (2)	7.2 (31)	21.7 (10)	> 0.999, 0.190	> 0.999, 0.600
12 months of follow‐up	34.6 (9)	15.5 (48)	35.7 (15)	0.025, > 0.999	0.060, > 0.999	11.5 (3)	0.0 (0)	16.7 (7)	> 0.999, 0.730	> 0.999, 0.913
Upper GI symptoms										
Hunger pain										
Baseline	11.1 (3)	9.1 (46)	32.8 (20)	0.728, 0.038	0.824, 0.253	7.4 (2)	5.1 (26)	19.7 (12)	0.645, 0.211	0.744, 0.603
1 month of follow‐up	3.8 (1)	4.3 (20)	20.0 (11)	> 0.999, 0.091	> 0.999, 0.420	3.8 (1)	1.5 (7)	5.5 (3)	0.350, > 0.999	0.553, > 0.999
6 months of follow‐up	18.5 (5)	4.9 (21)	23.9 (11)	0.014, 0.071	0.044, 0.355	11.1 (3)	1.6 (7)	13.0 (6)	0.017, > 0.999	0.064, > 0.999
12 months of follow‐up	26.9 (7)	5.9 (18)	23.8 (10)	0.001, 0.781	0.040, > 0.999	15.4 (4)	1.3 (4)	11.9 (5)	0.002, 0.723	0.017, 0.923
Nausea										
Baseline	29.6 (8)	28.8 (145)	41.0 (25)	> 0.999, 0.349	> 0.999, 0.838	14.8 (4)	14.7 (74)	21.3 (13)	> 0.999, 0.569	> 0.999, 0.875
1 month of follow‐up	11.5 (3)	8.5 (39)	10.9 (6)	0.482, > 0.999	0.578, > 0.999	7.7 (2)	0.8 (4)	5.5 (3)	0.034, 0.654	0.107, 0.957
6 months of follow‐up	25.9 (7)	7.5 (32)	17.8 (8)	0.005, 0.550	0.027, > 0.999	7.4 (2)	2.1 (9)	6.7 (3)	0.134, > 0.999	0.309, > 0.999
12 months of follow‐up	19.2 (5)	6.5 (20)	21.4 (9)	0.035, > 0.999	0.075, > 0.999	11.5 (3)	1.3 (4)	7.1 (3)	0.012, 0.668	0.051, 0.954
Heartburn										
Baseline	3.7 (1)	10.4 (53)	31.1 (19)	0.505, 0.005	0.594, 0.150	0.0 (0)	5.1 (26)	16.4 (10)	0.634, 0.028	0.764, 0.560
1 month of follow‐up	15.4 (4)	7.1 (33)	21.1 (12)	0.125, 0.765	0.208, > 0.999	7.7 (2)	2.3 (11)	12.3 (7)	0.143, 0.713	0.306, 0.972
6 months of follow‐up	29.6 (8)	10.0 (43)	39.1 (18)	0.006, 0.458	0.028, 0.859	11.1 (3)	3.3 (14)	15.2 (7)	0.072, 0.735	0.206, 0.900
12 months of follow‐up	30.8 (8)	9.8 (30)	35.7 (15)	0.005, 0.794	0.025, > 0.999	15.4 (4)	2.0 (6)	11.9 (5)	0.004, 0.723	0.022, 0.923
Acid regurgitation										
Baseline	3.7 (1)	7.5 (38)	27.9 (17)	0.711, 0.009	0.820, 0.135	0.0 (0)	4.2 (21)	9.8 (6)	0.617, 0.171	0.756, 0.641
1 month of follow‐up	24.0 (6)	7.0 (32)	19.3 (11)	0.009, 0.768	0.036, > 0.999	12.0 (3)	1.5 (7)	8.8 (5)	0.011, 0.695	0.051, 0.970
6 months of follow‐up	40.7 (11)	10.7 (46)	28.3 (13)	< 0.001, 0.310	0.015, 0.886	18.5 (5)	2.6 (11)	10.9 (5)	0.001, 0.483	0.050, 0.906
12 months of follow‐up	34.6 (9)	11.0 (34)	33.3 (14)	0.002, > 0.999	0.012, > 0.999	23.1 (6)	2.9 (9)	9.5 (4)	< 0.001, 0.165	0.012, 0.660
Eructation										
Baseline	11.1 (3)	11.0 (56)	24.6 (15)	> 0.999, 0.251	> 0.999, 0.793	7.4 (2)	5.3 (27)	8.2 (5)	0.651, > 0.999	0.737, > 0.999
1 month of follow‐up	15.4 (4)	6.1 (28)	22.8 (13)	0.082, 0.563	0.149, 0.994	0.0 (0)	1.1 (5)	7.0 (4)	> 0.999, 0.304	> 0.999, 0.760
6 months of follow‐up	18.5 (5)	10.9 (47)	28.3 (13)	0.216, 0.411	0.324, 0.881	7.4 (2)	3.7 (16)	10.9 (5)	0.288, > 0.999	0.480, > 0.999
12 months of follow‐up	23.1 (6)	9.7 (30)	23.8 (10)	0.046, > 0.999	0.095, > 0.999	3.8 (1)	2.3 (7)	9.5 (4)	0.480, 0.642	0.640, 0.963
Lower GI symptoms										
Diarrhea										
Baseline	59.3 (16)	36.9 (187)	47.5 (29)	0.025, 0.360	0.058, 0.831	48.1 (13)	22.3 (113)	32.8 (20)	0.004, 0.233	0.024, 0.635
1 month of follow‐up	23.1 (6)	6.9 (32)	12.3 (7)	0.011, 0.328	0.041, 0.820	3.8 (1)	1.3 (6)	1.8 (1)	0.314, 0.531	0.509, 0.861
6 months of follow‐up	40.7 (11)	9.1 (39)	17.4 (8)	< 0.001, 0.051	0.012, 0.306	18.5 (5)	2.1 (9)	4.3 (2)	< 0.001, 0.093	0.015, 0.558
12 months of follow‐up	38.5 (10)	6.5 (20)	31.0 (13)	< 0.001, 0.602	0.010, 0.926	15.4 (4)	1.6 (5)	9.5 (4)	0.003, 0.470	0.020, 0.910
Loose stool										
Baseline	48.1 (13)	25.0 (127)	41.0 (25)	0.012, 0.624	0.042, 0.936	37.0 (10)	12.8 (65)	27.9 (17)	0.002, 0.455	0.017, 0.941
1 month of follow‐up	15.4 (4)	7.8 (36)	5.3 (3)	0.257, 0.198	0.367, 0.743	3.8 (1)	1.1 (5)	1.8 (1)	0.276, 0.531	0.473, 0.885
6 months of follow‐up	40.7 (11)	7.2 (31)	23.9 (11)	< 0.001, 0.187	0.009, 0.748	22.2 (6)	2.8 (12)	4.3 (2)	< 0.001, 0.045	0.015, 0.175
12 months of follow‐up	38.5 (10)	6.5 (20)	35.7 (15)	< 0.001, > 0.999	0.008, > 0.999	19.2 (5)	1.6 (5)	7.1 (3)	< 0.001, 0.244	0.020, 0.637
Urgent need for defecation										
Baseline	34.6 (9)	15.0 (76)	21.3 (13)	0.013, 0.281	0.043, 0.843	26.9 (7)	8.7 (44)	13.1 (8)	0.008, 0.132	0.040, 0.660
1 month of follow‐up	15.4 (4)	3.9 (18)	17.5 (10)	0.024, > 0.999	0.060, > 0.999	7.7 (2)	0.6 (3)	10.5 (6)	0.024, > 0.999	0.085, > 0.999
6 months of follow‐up	18.5 (5)	4.0 (17)	19.6 (9)	0.006, > 0.999	0.028, > 0.999	7.4 (2)	2.1 (9)	10.9 (5)	0.133, > 0.999	0.333, > 0.999
12 months of follow‐up	19.2 (5)	5.5 (17)	23.8 (10)	0.020, 0.769	0.055, > 0.999	11.5 (3)	2.6 (8)	14.3 (6)	0.045, > 0.999	0.135, > 0.999
Constipation										
Baseline	14.8 (4)	9.0 (46)	23.3 (14)	0.304, 0.568	0.397, 0.947	0.0 (0)	4.1 (21)	15.0 (9)	0.617, 0.052	0.756, 0.520
1 month of follow‐up	23.1 (6)	10.6 (49)	33.3 (19)	0.101, 0.443	0.178, 0.917	3.8 (1)	2.1 (10)	21.1 (12)	0.448, 0.054	0.625, 0.405
6 months of follow‐up	14.8 (4)	8.9 (38)	43.5 (20)	0.298, 0.019	0.397, 0.190	3.7 (1)	3.0 (13)	21.7 (10)	0.581, 0.046	0.726, 0.552
12 months of follow‐up	7.7 (2)	9.4 (29)	40.5 (17)	> 0.999, 0.005	> 0.999, 0.150	3.8 (1)	1.9 (6)	21.4 (9)	0.435, 0.076	0.621, 0.507
Hard stool										
Baseline	14.8 (4)	7.9 (40)	18.0 (11)	0.267, > 0.999	0.373, > 0.999	0.0 (0)	3.2 (16)	13.1 (8)	> 0.999, 0.100	> 0.999, 0.545
1 month of follow‐up	26.9 (7)	10.2 (47)	28.1 (16)	0.017, > 0.999	0.049, > 0.999	7.7 (2)	2.5 (12)	15.8 (9)	0.162, 0.489	0.324, 0.889
6 months of follow‐up	14.8 (4)	9.1 (39)	41.3 (19)	0.308, 0.021	0.393, 0.180	7.4 (2)	3.3 (14)	21.7 (10)	0.245, 0.190	0.432, 0.633
12 months of follow‐up	12.0 (3)	11.0 (34)	40.5 (17)	0.748, 0.015	0.831, 0.180	8.0 (2)	3.2 (10)	21.4 (9)	0.224, 0.189	0.407, 0.667
Feeling of incomplete evacuation										
Baseline	22.2 (6)	9.7 (49)	23.0 (14)	0.049, > 0.999	0.092, > 0.999	11.1 (3)	4.4 (22)	9.8 (6)	0.127, > 0.999	0.331, > 0.999
1 month of follow‐up	15.4 (4)	6.7 (31)	19.3 (11)	0.108, 0.767	0.185, > 0.999	3.8 (1)	1.9 (9)	15.8 (9)	0.418, 0.160	0.627, 0.686
6 months of follow‐up	22.2 (6)	7.0 (30)	41.3 (19)	0.014, 0.128	0.042, 0.549	7.4 (2)	2.3 (10)	21.7 (10)	0.156, 0.190	0.323, 0.633
12 months of follow‐up	15.4 (4)	7.6 (23)	31.0 (13)	0.250, 0.249	0.366, 0.830	7.7 (2)	1.6 (5)	16.7 (7)	0.098, 0.465	0.267, 0.930

*Note*: All results are presented as % (*n*). Post‐COVID‐19 DGBIs group consists of patients with COVID‐19 inflammation who met criteria for DGBIs during follow‐up based on Rome IV criteria. Non‐DGBI group consists of patients with COVID‐19 inflammation who did not meet DGBI criteria at any point during the follow‐up period. The pre‐existing IBS/FD group consists of patients with COVID‐19 inflammation and a baseline diagnosis of IBS or FD based on Rome IV criteria.

Abbreviations: DGBI, disorders of gut‐brain interaction; FD, functional dyspepsia; FDR, false discovery rate; GI, gastrointestinal; IBS, irritable bowel syndrome; *n*, number.

^a^ Presence of GI symptoms was evaluated on the GSRS using a cutoff that distinguished “no discomfort at all” from all other levels (“very mild,” “mild,” “moderate,” “moderate‐severe,” “severe,” and “very severe”).

^b^ Clinically significant symptoms were assessed on the GSRS using a cutoff that grouped “no discomfort at all,” “very mild,” and “mild” together versus “moderate,” “moderate‐severe,” “severe,” and “very severe”.

^c^ *p*‐values for comparisons between post‐COVID‐19 DGBIs and non‐DGBI controls.

^d^ *p*‐values for comparisons between post‐COVID‐19 DGBIs and patients with pre‐existing IBS/FD.

In the post‐COVID‐19 DGBI group, the prevalence rates of all abdominal symptoms and most upper GI symptoms (e.g., hunger pain, heartburn, acid regurgitation, and eructation) increased progressively over time, with the most substantial increases observed at 6 and 12 months of follow‐up compared to baseline. In the pre‐existing IBS/FD group, GI symptoms decreased over time, with lower GI symptoms having a more pronounced decline compared to the post‐COVID‐19 DGBI group. In the non‐DGBI group, symptom prevalence remained consistently low across all time points (Table 3).

As expected, the crude odds of experiencing each of the assessed GI symptoms were significantly elevated in patients with post‐COVID‐19 DGBIs compared with non‐DGBI controls, except for abdominal distension and borborygmus (Table 4). The crude odds of experiencing each assessed GI symptom in patients with post‐COVID‐19 DGBIs compared to those with pre‐existing IBS/FD are presented in Supporting Information S1: Table S1.

**TABLE 4:** Crude and adjusted odds ratios for GI symptoms over a 1‐year follow‐up period following COVID‐19 infection among post‐COVID‐19 DGBI group compared to non‐DGBI group.

GSRS symptoms	Crude^a^ OR *p*‐value, FDR	Adjusted OR for time *p*‐value^b^, FDR	Adjusted OR for time, depression at 6 months and gender *p*‐value^b^, FDR	Adjusted for time, depression at 12 months and gender *p*‐value^b^, FDR	Adjusted for time, anxiety at 6 months and gender *p*‐value^b^, FDR	Adjusted for time, anxiety at 12 months and gender^b^, FDR
Abdominal symptoms						
Abdominal pain	3.99	4.38	4.41	4.46	3.49	4.31
	< 0.001, < 0.001	< 0.001, < 0.001	< 0.001, < 0.001	< 0.001, < 0.001	< 0.001, 0.001	< 0.001, < 0.001
Abdominal distention	2.68	2.62	2.36	2.32	1.72	2.140
	< 0.001, < 0.001	0.001, 0.004	0.010, 0.019	0.013, 0.028	0.138, 0.231	0.028, 0.060
Borborygmus	1.59	1.59	1.37	1.38	0.85	1.17
	0.066, 0.066	0.184, 0.197	0.414, 0.414	0.405, 0.434	0.681, 0.729	0.673, 0.673
Increased flatus	2.30	2.25	1.99	1.86	1.27	1.53
	< 0.001, < 0.001,	0.008, 0.014	0.033, 0.049	0.060, 0.090	0.503, 0.629	0.221, 0.301
Upper GI symptoms						
Hunger pain	2.71	2.74	3.03	2.58	2.19	2.35
	< 0.001, < 0.001	0.002, 0.004	0.003, 0.007	0.007, 0.019	0.061, 0.131	0.018, 0.044
Nausea	1.72	1.96	2.08	2.21	1.59	2.02
	0.027, 0.031	0.106, 0.122	0.071, 0.088	0.044, 0.073	0.262, 0.357	0.083, 0.139
Heartburn	2.41	2.38	2.82	2.28	2.70	2.11
	0.001, 0.001	0.010, 0.015	0.003, 0.007	0.025, 0.047	0.015, 0.037	0.054, 0.102
Acid regurgitation	3.58,	3.49	4.47	3.59	3.60	3.41
	< 0.001, < 0.001	< 0.001, 0.001	< 0.001, < 0.001	< 0.001, 0.002	< 0.001, 0.004	0.001, 0.002
Eructation	1.96	1.95	1.52	1.52	1.03	1.43
	0.013, 0.016	0.071, 0.097	0.272, 0.292	0.280, 0.323	0.945, 0.945	0.377, 0.435
Lower GI symptoms						
Diarrhea	3.51	4.86	4.50	4.38	3.75	4.48
	< 0.001, < 0.001	< 0.001, < 0.001	< 0.001, < 0.001	< 0.001, < 0.001	< 0.001, 0.001	< 0.001, < 0.001
Loose stool	3.89	4.68	4.14	3.88	3.24	3.69
	< 0.001, < 0.001	< 0.001, < 0.001	< 0.001, < 0.001	< 0.001, 0.001	0.001, 0.005	< 0.001, 0.002
Urgent need for defecation	3.46	3.94	3.66	3.34	2.95	3.25
	< 0.001, < 0.001	< 0.001, < 0.001	0.001, 0.003	0.003, 0.008	0.010, 0.029	0.003, 0.008
Constipation	1.69	1.71	1.66	1.42	1.32	1.46
	0.063, 0.066	0.221, 0.221	0.267, 0.292	0.457, 0.457	0.587, 0.678	0.433, 0.464
Hard stool	1.99	1.98	2.16	1.64	1.67	1.58
	0.011, 0.015	0.096, 0.120	0.064, 0.087	0.255, 0.319	0.262, 0.357	0.301, 0.376
Feeling of incomplete evacuation	2.73	2.77	2.51	1.93	1.87	1.76
	< 0.001, < 0.001	0.004, 0.008	0.012, 0.021	0.083, 0.113	0.125, 0.231	0.147, 0.221

Abbreviations: DGBI, disorders of gut‐brain interaction; FDR, false discovery rate; OR, odds‐ratio.

^a^ Calculated by univariate logistic regression.

^b^ Calculated by generalized estimating equation (GEE) for repeated measures.

To investigate potential factors contributing to the observed increase in GI symptoms among patients with post‐COVID‐19 DGBIs during follow‐up, a comparison of chronic medication intake with potential GI effects was conducted between the study groups (Supporting Information S1: Table S2), showing no significant differences within groups.

### 4.4 Severity of GI Symptoms

Patients with post‐COVID‐19 DGBIs demonstrated significantly lower scores for symptom severity throughout the study period compared to patients with pre‐existing IBS/FD. Specifically, patients with post‐COVID‐19 DGBIs reported significant less severe abdominal pain (1.8 ± 1.2 vs. 2.1 ± 1.6, *p* = 0.017, FDR = 0.068), hunger pains (1.4 ± 1.0 vs. 1.7 ± 1.3, *p* = 0.028, FDR = 0.028), heartburn (1.5 ± 1.0 vs. 1.8 ± 1.4, *p* = 0.026, FDR = 0.035), and abdominal distention (1.6 ± 1.1 vs. 2.2 ± 1.6, *p* = 0.021, FDR = 0.042).

Symptom trajectories of patients with post‐COVID‐19 DGBI were different when compared with non‐DGBI controls. Patients with post‐COVID‐19 DGBIs experienced a significant increase over time in the severity of abdominal pain (*p* for trend = 0.032, FDR = 0.080), hunger pain (*p* for trend = 0.010, FDR = 0.038), heartburn (*p* for trend < 0.001, FDR = 0.001), and acid regurgitation (*p* for trend < 0.001, FDR < 0.001), while a significant improvement was observed in the severity of diarrhea (*p* for trend < 0.001, FDR = 0.001) and urgency (*p* for trend = 0.024, FDR = 0.072). Non‐DGBI controls showed either gradual improvement or symptom stability, in contrast with the trends observed in patients with post‐COVID‐19 DGBIs. This divergence resulted in a significant interaction between the two groups over time in the severity of abdominal pain (*p* for interaction < 0.001, FDR = 0.001), hunger pain (*p* for interaction = 0.001, FDR = 0.004), heartburn (*p* for interaction < 0.001, FDR = 0.001), and acid regurgitation (*p* for interaction < 0.001, FDR < 0.001) (Figure 2). Similar differences in symptom trajectories were found when compared with patients with pre‐existing IBS/FD were assessed. While the severity of abdominal pain, hunger pain, and acid regurgitation increased over time among patients with post‐COVID‐19 DGBI, it decreased or remained stable in patients with pre‐existing IBS/FD (Figure 3).

FIGURE 2Fluctuation over time of GI symptoms complained by patients with post‐COVID‐19 DGBIs and non‐DGBIs controls, as evaluated using the GSRS questionnaire. The GSRS assesses 15 GI symptoms rated on a 1–7 severity scale, where “1” indicates “no discomfort at all,” “2” corresponds to “very mild,” “3” to “mild,” “4” to “moderate,” “5” to “moderate‐severe,” “6” to “severe,” and “7” to “very severe.” (2A) Fluctuation over time of abdominal symptoms. (2B) Fluctuation over time of upper gastrointestinal symptoms. (2C) Fluctuation over time of lower gastrointestinal symptoms.

**Figure:**
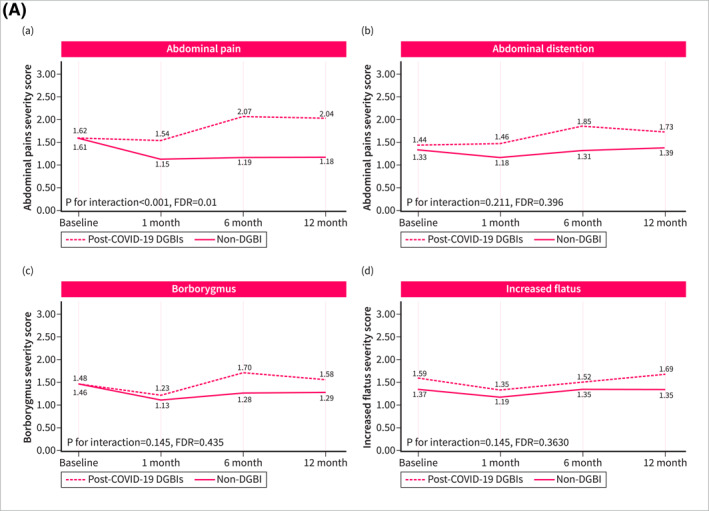

**Figure:**
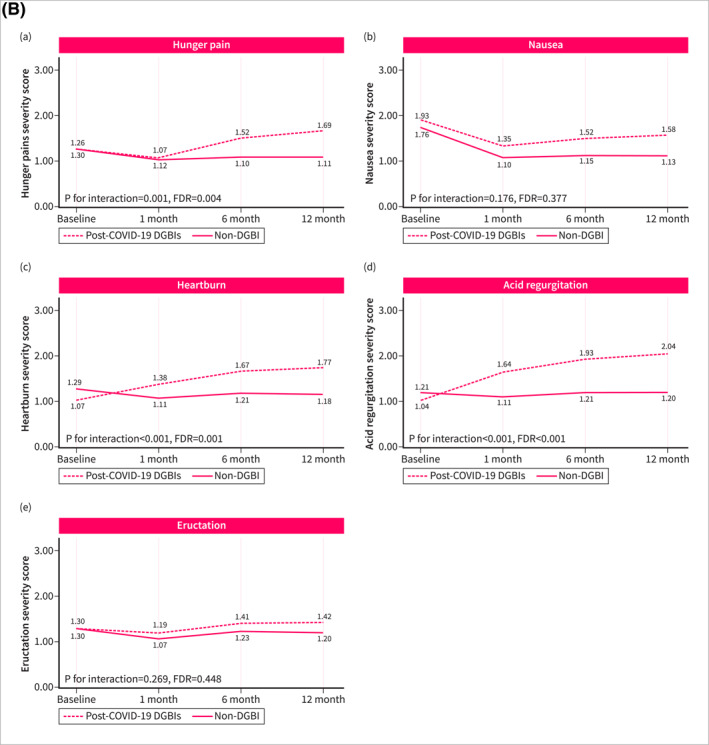

**Figure:**
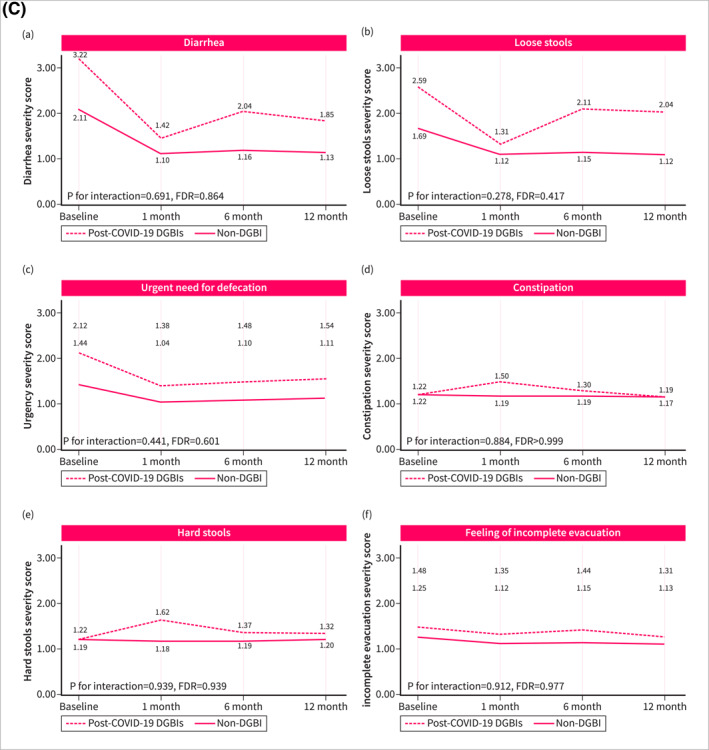

FIGURE 3Fluctuation over time of GI symptoms complained by patients with post‐COVID‐19 DGBIs and pre‐existing IBS/FD controls, as evaluated using the GSRS questionnaire. The GSRS assesses 15 GI symptoms rated on a 1–7 severity scale, where “1” indicates “no discomfort at all,” “2” corresponds to “very mild,” “3” to “mild,” “4” to “moderate,” “5” to “moderate‐severe,” “6” to “severe,” and “7” to “very severe.” (2A) Fluctuation over time of abdominal symptoms. (2B) Fluctuation over time of upper gastrointestinal symptoms. (2C) Fluctuation over time of lower gastrointestinal symptoms.

**Figure:**
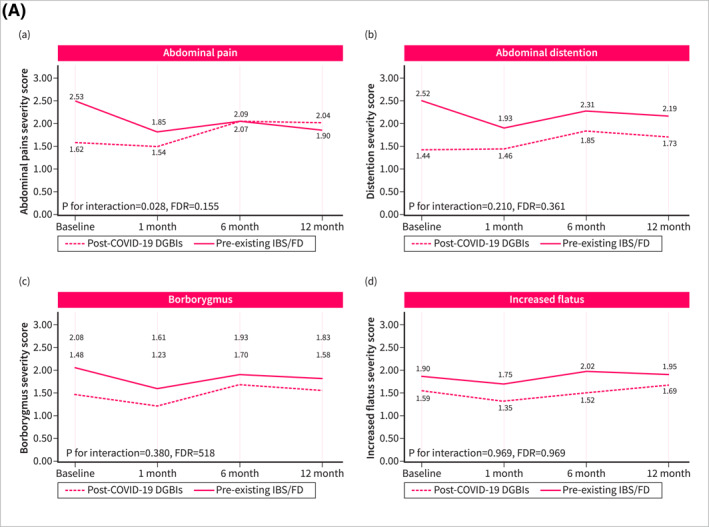

**Figure:**
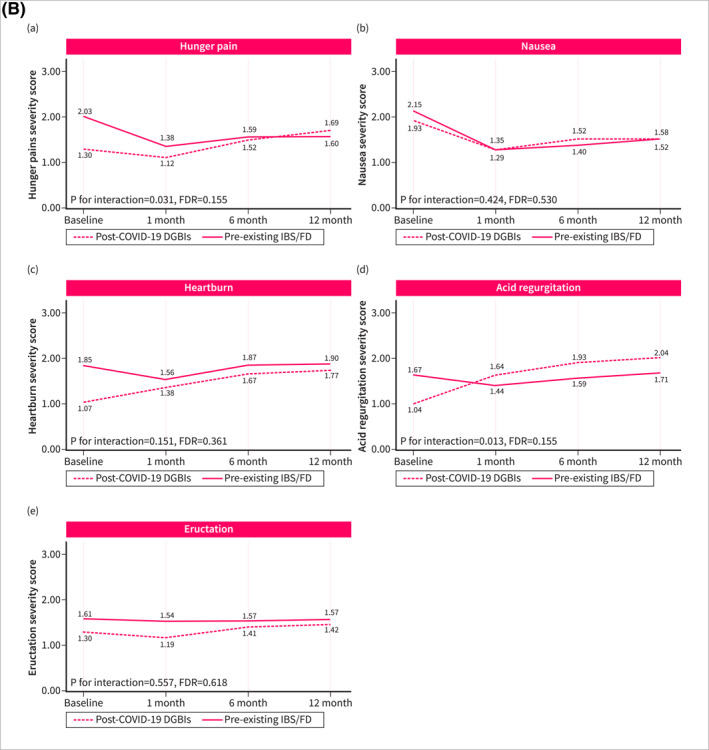

**Figure:**
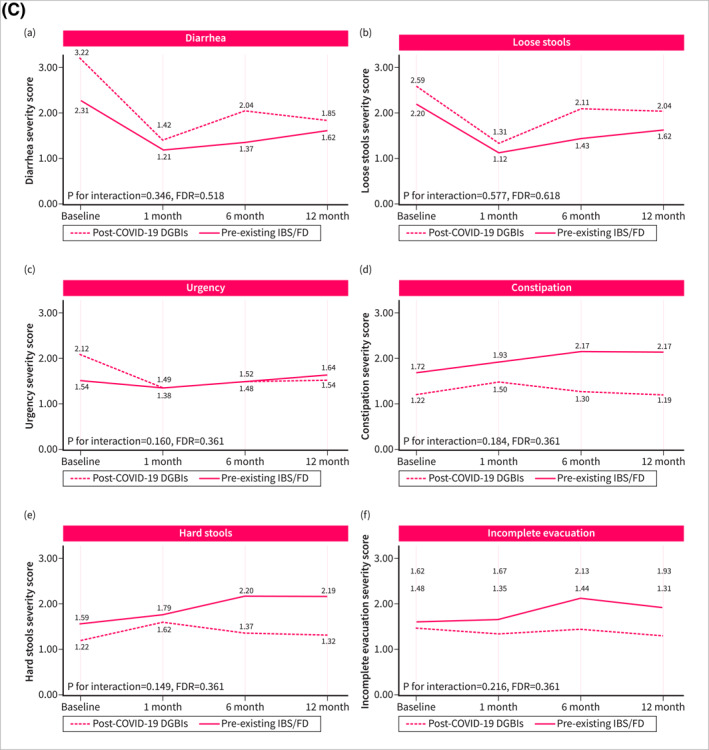

Although there were statistically significant differences in GI symptom severity, most of the severity scores reported for GI symptoms remained below a GSRS score of 3 (mild discomfort). Focusing on clinically significant symptoms (GSRS score > 3, Table 3), we found significant differences primarily in the comparison between the post‐COVID‐19 DGBI group and the non‐DGBI control group for most GI symptoms during the second phase of the time frame (6–12 months).

## 5 Discussion

This post hoc analysis of the original GI‐COVID study [7] aimed to investigate the changes in GI and psychological symptoms over 1 year after hospital discharge in COVID‐19 patients developing post‐COVID‐19 DGBIs, compared with a group of patients with pre‐existing IBS/FD and a group of non‐DGBI controls. In our previous study, we assessed the prevalence of post‐COVID‐19 DGBIs following hospitalization for SARS‐CoV‐2 infection compared to COVID‐19 negative controls, and we observed a higher incidence of Rome IV IBS within this cohort relative to hospitalized patients who tested negative for COVID‐19 (3.2% vs. 0.5%, *P* = 0.045) [7]. However, we excluded patients with IBS or FD diagnosis before COVID‐19 in order to assess the true incidence of newly incident diagnoses of DGBIs. In our recently published meta‐analysis, which sought to estimate the prevalence of post‐COVID‐19 FD and IBS, we observed that COVID‐19 survivors demonstrated an increased risk of developing IBS compared with controls (OR = 6.27, 95% CI: 0.88–44.76, *p* = 0.067, *I* ^2^ = 81.4%) [8]. However, in that analysis, we excluded all patients with pre‐existing chronic GI disorders, distinguishing it from the present study.

The impact of the COVID‐19 pandemic on patients with DGBIs was reported by Oshima et al. [16] in an online survey including 1092 patients with IBS and FD compared with 4065 control patients. Patients with FD‐only, IBS‐only, and FD–IBS overlap reported a significant symptoms worsening than those in the non‐FD/IBS group (respectively 19.6%, 31.9% and 50.4% vs. 6.4%, *p* < 0.001) [12]. However, the authors did not explore the SARS‐CoV2 status of patients, making it unclear whether symptoms worsened due to increased psychological factors or COVID‐19 infection itself. Similarly, another study used a cross‐sectional design to examine the effect of the COVID‐19 pandemic on GI symptoms and psychological status of patients with IBS, reporting worsened bowel habits across all IBS subtypes [11]. This study, however, did not assess the SARS‐CoV2 status of patients. Gubatan et al. [12] evaluated in a monocentric retrospective study COVID‐19 inpatients and outpatients with IBS, gastroparesis, or FD and found significantly more GI symptoms 6 months after the acute bout of COVID‐19 infection compared with those reported 6 months before, although these latter were assessed with a significant recall bias.

Our manuscript reports on prospectively collected data using strict inclusion criteria and validated questionnaires. We found that the rate of patients who developed post‐COVID‐19 DGBI was consistent with other experiences [8, 17]. Patients with post‐COVID‐19 DGBI showed an increased prevalence of GI symptoms over time and experienced progressive worsening of abdominal pain, hunger pain, heartburn, and acid regurgitation, in line with a recent meta‐analysis reporting an increasing prevalence of symptoms and DGBIs diagnosis up to 1 year and after an acute bout of infection [18]. In contrast, our results are in contrast with a recently published retrospective Spanish experience not using strict inclusion criteria, reporting that the prevalence of overall gastrointestinal post‐COVID symptoms and diarrhea decreased over time [19].

We also found that patients with pre‐existing IBS/FD with confirmed SARS‐CoV2 infection had the highest prevalence of clinically significant symptoms during follow‐up, which improved over time with the exception of constipation, hard stool and incomplete evacuation, which worsened over time. The baseline difference in symptoms reported by the different groups is consistent with a possible susceptibility for developing post‐COVID‐19 DGBIs and in the group with pre‐existing IBS/FD with the chronic nature of DGBIs. Instead, the significantly higher values of GSRS scores at follow‐up, which are markers of symptom severity, may suggest both a greater long‐term effect of COVID‐19 inflammation and a greater psychological burden compared with non‐DGBI controls. Symptoms worsening over time was significant for constipation, hard stools, and feeling of incomplete evacuation among the pre‐existing IBS/FD group, even when evaluating possible differences among groups for the chronic use of drugs possibly influencing these GI symptoms. These results are in line with a recent meta‐analysis reporting that 31.4% (95% CI, 15.9–52.5) of patients with DGBI experienced symptom deterioration during the COVID‐19 pandemic [20]. Interestingly, the increased rates and severity of constipation in patients with pre‐existing IBS/FD are consistent with previous findings [21]. In fact, although not specifically assessed in patients with IBS/FD, a broad characterization of post‐acute sequelae of COVID‐19 showed significantly higher rates of constipation 6 months after COVID‐19, which were proportional to the severity of the acute event [21].

Regarding the effect of the infection on anxiety and depression, we reported significantly higher HADS scores in patients with post‐COVID‐19 DGBISs and with pre‐existing IBS/FD than non‐DGBI controls at 6 months, similar to data reported in other studies [11, 16, 22] suggesting a considerable effect of psychological comorbidities on symptoms persistence.

Notably, our results on GI symptoms trends were adjusted both for time and, above all, for anxiety and depression. Therefore, we speculate that these findings can be directly associated with the effect of SARS‐CoV2 infection and not dependent on the psychological effects associated with the COVID‐19 pandemic. Moreover, in line with global trends [23], the rate of females in the post‐COVID‐19 DGBI and the pre‐existing IBS/FD group was higher than the that in the control group. Nevertheless, after adjusting for gender, our results remained significant.

In addition, while we confirmed data from previous studies reporting the occurrence of post‐COVID‐19 DGBIs together with increased levels of anxiety and depression in previously healthy subjects [8], we highlighted differing trajectories of GI symptoms and the distinct impact of COVID‐19 on patients with pre‐existing IBS/FD versus post‐COVID‐19 DGBIs.

It should be underlined that several translational studies support a direct and persistent effect of SARS‐CoV2 on GI physiology. SARS‐CoV2 can infect the GI tract, by binding the Angiotensin‐Converting Enzyme‐2 receptors, while active viral replication was described on electron microscopy of intestinal biopsies [24]. Other studies have also found persistently active immunological cells on intestinal biopsies several months after an initial SARS‐CoV‐2 infection [25]. Moreover, significant alterations in the gut microbiota caused by COVID‐19 were found both during the acute phase and after 1 year from the initial infection [24]. SARS‐CoV‐2 antigen persistence in the GI tract may contribute to secretomotor dysfunction and symptom perception by affecting enteric and afferent nerves, leading to increased symptomatology [26]. Recently, more evidence on post‐infection IBS has demonstrated durable alterations in serotonergic signaling and 5‐ hydroxytryptamine (5‐HT) processing, likely due to underlying dysbiosis, leading to alterations in gut motility, secretions, and sensitivity [27]. This evidence fits in a wider context of IBS as a disease characterized by chronic structural, microbial, and immunological alterations which can be further modified by environmental hits. The present post hoc analysis suggests that those patients developing post‐COVID‐19 DGBIs experience an increasing symptom burden and progressive deterioration over time when compared with other groups. Furthermore, COVID‐19 may serve as an additional factor contributing to symptom worsening and persistence in patients with pre‐existing IBS/FD [27].

The main strength of our study relies on its prospective design analyzing the effects of an acute event, specifically severe COVID‐19 infection, able to trigger the onset of newly diagnosed DGBIs and to modify the course of chronic diseases, such as IBS or FD. Our analysis shows that patients with post‐COVID‐19 DGBIs as well as those with pre‐existing IBS/FD experience not only an exacerbation of GI symptoms compared with healthy controls but also report a greater overall psychological burden. Moreover, our results also have significant clinical relevance, since we found that the pre‐existing IBS/FD group showed a greater proportion of clinically significant GI symptoms (GSRS > 3) in all time points.

An additional strength of our study is its multicenter design, which involved multiple sites across various regions. This approach enhances the external validity of our findings, as it allows for a more diverse patient population and increases the generalizability of the results to broader settings. By incorporating data from multiple centers, we reduce the potential bias associated with single‐center studies, making our conclusions more robust and applicable to different patient populations.

Our investigation has significant limitations. First, our results may have been impacted by the limited 1‐year time follow‐up period. However, we believe that our findings have a biological and epidemiological plausibility since a recent study including patients with COVID‐19 infection without any previous DGBI reported that GI symptoms following COVID‐19 infection can persist for more than one year after the acute bout of infection [28]. A potential source of bias is the choice of conducting interviews and administering surveys over the telephone during the follow‐up period, even though the questionnaire was originally designed to be self‐administered. Furthermore, the diagnoses of IBS and FD were established prior to the study using Rome IV criteria. While we relied on the accuracy and completeness of the medical records and could not independently verify that all relevant diagnostic tests were consistently conducted for every patient, these diagnoses were made by specialists at tertiary centers. Therefore, we believe that they inherently adhered to standard clinical practices, including the necessary diagnostic processes. Additionally, the GSRS data available for the pre‐baseline period only captured the presence or absence of GI symptoms with dichotomous questions, rather than the severity score of these symptoms. This contrasts with the data collected at all other time points in the study, where the GSRS was assessed using a severity score, making it impossible to quantitatively evaluate changes in symptom severity relative to the pre‐baseline period. Consequently, caution should be exercised in interpreting the findings, as the lack of detailed severity data before baseline limits the ability to determine whether GI symptoms worsened due to COVID‐19 or simply returned to their actual pre‐baseline levels. Moreover, the baseline questionnaire may also be affected by a recall bias. Because of the timing of the study, it was not possible to consider significant factors such as the emergence of SARS‐CoV2 genetic variations and, more significantly, the substantial influence of widespread immunization against the virus. Moreover, it remains unclear whether multiple COVID‐19 episodes might further influence the natural history of patients with post‐COVID‐19 DGBIs and those with pre‐existing IBS or FD.

Future studies with larger sample sizes should aim to analyze IBS and FD as distinct entities to provide deeper insights into their unique responses to COVID‐19 infection. Moreover, incorporating a control group of patients with pre‐existing IBS/FD who have not had COVID‐19 infection would be essential to clarify the role of COVID‐19 in exacerbating symptoms. Such a design would enable the evaluation of the natural trajectory of these GI disorders and further clarify whether symptom progression is directly attributable to COVID‐19.

In conclusion, patients hospitalized for COVID‐19 developing post‐COVID‐19 DGBIs as well as those with pre‐existing IBS/FD exhibit more severe GI symptoms compared with healthy individuals after one year from the acute bout of infection, together with greater psychological distress over time. Overall, most GI and psychological symptoms decrease over time after SARS‐CoV2 infection. However, while patients with pre‐existing IBS/FD hospitalized for COVID‐19 had more clinically significant symptoms but were more stable over time, patients with post‐COVID‐19 DGBIs experienced a significant worsening.

## Author Contributions

**Giovanni Marasco:** supervised, designed the study, collected and interpreted data, and drafted the manuscript. **Keren Hod:** designed the study, collected and interpreted data, performed statistical analysis, and drafted the manuscript. **Giovanni Barbara:** supervised, critically revised and approved the final version of the draft. All authors collected data for the study and critically revised and approved the version of the manuscript.

## Ethics Statement

This study involved human participants and was approved by IRCCS Policlinico S. Orsola Ethical Committee—Coordinating center approval: 399/2020/Oss/AOUBo. Participants gave informed consent to participate in the study before taking part.

## Consent

Written informed consent was obtained from all study participants.

## Conflicts of Interest

The authors declare no conflicts of interest.

## Permission to Reproduce Material From Other Sources

Not applicable.

## Supporting information

Supporting Information S1

Figure S1

## Data Availability

Data are available upon reasonable request. All figures have associated raw data. Additional data that support the findings of this study are available from the corresponding author upon request.
