# Supplementary material for: Long‐Term Impact of COVID‐19 on Disorders of Gut–Brain Interaction: Incidence, Symptom Burden, and Psychological Comorbidities
Source: United European Gastroenterol J. 2025 Mar 21;13(5):798–818. doi: 10.1002/ueg2.70005 (PMC12188378; doi:10.1002/ueg2.70005)
Supplement: Supplementary file 1 — Supporting Information S1 [file UEG2-13-798-s002.docx]

**Supplementary Table 1**. Crude and adjusted odds ratios for GI symptoms over a 1-year follow-up period following COVID-19 infection among post-COVID-19 DGBI group compared to pre-existing IBS/FD group

| **Adjusted for time, anxiety at 12 months and gender^2^, FDR** | **Adjusted for time, anxiety at 6 months and gender**  **p-value^2^, FDR** | **Adjusted for time, depression at 12 months and gender**  **p-value^2^, FDR** | **Adjusted OR**  **for time, depression at 6 months and gender**  **p-value^2^, FDR** | **Adjusted OR**  **for time**  **p-value^2^, FDR** | **Crude^1^**  **OR**  **p-value, FDR** | **GSRS symtoms** |
| --- | --- | --- | --- | --- | --- | --- |
|  |  |  |  |  |  | **Abdominal symptoms** |
| 0.96  0.910, >0.999 | 0.98  0.963, >0.999 | 0.96  0.910, >0.999 | 1.08  0.830, 0.830 | 0.85  0.635, 0.794 | 0.86  0.560, 0.700 | Abdominal pain |
| 0.42  0.043, 0.323 | 0.46  0.073, 0.548 | 0.42  0.042, 0.315 | 0.55  0.130, 0.975 | 0.48  0.041, 0.308 | 0.49  0.006, 0.045 | Abdominal distention |
| 0.44  0.060, 0.300 | 0.43  0.074, 0.370 | 0.44  0.077, 0.289 | 0.54  0.165, 0.619 | 0.50  0.093, 0.199 | 0.51  0.019, 0.071 | Borborygmus |
| 0.63  0.232, 0.387 | 0.66  0.303, 0.568 | 0.63  0.236, 0.393 | 0.75  0.453, 0.618 | 0.64  0.212, 0.353 | 0.66  0.125, 0.170 | Increased flatus |
|  |  |  |  |  |  | **Upper GI symptoms** |
| 0.65  0.309, 0.464 | 0.66  0.354, 0.590 | 0.65  0.313, 0.470 | 0.72  0.449, 0.674 | 0.51  0.080, 0.300 | 0.52  0.038, 0.081 | Hunger pain |
| 1.00  0.998, 0.998 | 1.06  0.907, >0.999 | 0.98  0.973, 0.973 | 1.38  0.517, 0.646 | 0.92  0.849, 0.849 | 0.89  0.699, 0.807 | Nausea |
| 0.56  0.164,0.492 | 0.70  0.395, 0.593 | 0.53  0.132, 0.396 | 0.69  0.351, 0.752 | 0.51  0.081, 0.243 | 0.55  0.036, 0.090 | Heartburn |
| 1.00  0.990, >0.999 | 1.26  0.592, 0.740 | 1.03  0.946, >0.999 | 1.22  0.639, 0.737 | 0.90  0.789, 0.910 | 0.95  0.852, 0.852 | Acid regurgitation |
| 0.54  0.207, 0.388 | 0.59  0.257, 0.551 | 0.53  0.189, 0.405 | 0.65  0.343, 0.858 | 0.61  0.251, 0.342 | 0.62  0.119, 0.179 | Eructation |
|  |  |  |  |  |  | **Lower GI symptoms** |
| 1.67  0.186, 0.399 | 1.64  0.197, 0.591 | 1.56  0.229, 0.429 | 1.75  0.131, 0.655 | 1.93  0.056, 0.280 | 1.78  0.021, 0.063 | Diarrhea |
| 1.32  0.498, 0.679 | 1.39  0.421, 0.574 | 1.31  0.516, 0.704 | 1.44  0.369, 0.692 | 1.61  0.224, 0.336 | 1.57  0.078, 0.146 | Loose stool |
| 1.15  0.761, 0.951 | 1.00  0.988, 0.988 | 1.18  0.733, 0.916 | 1.12  0.800, 0.857 | 1.10  0.819, 0.878 | 1.09  0.756, 0.810 | Urgent need for defecation |
| 0.29  0.030, 0.450 | 0.34  0.044, 0.660 | 0.25  0.019, 0.285 | 0.35  0.049, 0.735 | 0.33  0.027, 0.405 | 0.343,  <0.001, 0.008 | Constipation |
| 0.39  0.067, 0.251 | 0.50  0.170, 0.638 | 0.37  0.052,0.260 | 0.53  0.194, 0.582 | 0.45  0.090, 0.225 | 0.47  0.012, 0.060 | Hard stool |
| 0.54  0.168, 0.420 | 0.55  0.218,0.545 | 0.51  0.135, 0.338 | 0.67  0.374, 0.623 | 0.58  0.191, 0.358 | 0.61  0.089, 0.148 | Feeling of incomplete evacuation |

FDR, false discovery rate; OR, odds-ratio0.218

^1^Calculated by univariate logistic regression.

^2^Calculated by generalized estimating equation (GEE) for repeated measure

**Supplementary Table 2.** Comparison of chronic medication intake with possible GI effects between study groups.

| **Chronic medication intake with possible GI effects** | **Post-COVID-19 DGBIs (n=27)** | **Non-DGBI (n=511)** | **Pre-existing IBS/FD (n=61)** | **P value^1,2^** | **FDR^1,2^** |
| --- | --- | --- | --- | --- | --- |
| Proton pump inhibitors (PPI) | 18.5 (5) | 8.8 (45) | 37.7 (23) | 0.095, 0.087 | >0.999, 0.830 |
| Non-steroidal anti-inflammatory | 0.0 (0) | 4.3 (22) | 8.2 (5) | 0.618, 0.318 | >0.999, >0.999 |
| Steroids | 0.0 (0) | 1.4 (7) | 3.3 (2) | >0.999, >0.999 | >0.999, >0.999 |
| Metformin | 11.1 (3) | 4.9 (25) | 9.8 (6) | 0.160, 0.999 | >0.999, >0.999 |
| Selective serotonin reuptake inhibitors (SSRIs) | 0.0 (0) | 1.6 (8) | 4.9 (3) | >0.999, 0.550 | >0.999, >0.999 |
| Antipsychotic | 0.0 (0) | 0.6 (3) | 1.6 (1) | >0.999, >0.999 | >0.999, >0.999 |
| Iron | 0.0 (0) | 1.4 (7) | 0.0 (0) | >0.999, 0.999 | >0.999, >0.999 |
| Fibrates | 0.0 (0) | 1.2 (6) | 0.0 (0) | >0.999, >0.999 | >0.999, >0.999 |
| ACE inhibitors | 7.4 (2) | 9.6 (49) | 11.5 (7) | >0.999, 0.716 | >0.999, >0.999 |
| β-blockers | 11.1 (3) | 11.2 (57) | 16.4 (10) | >0.999, 0.747 | >0.999, >0.999 |
| Angiotensin-2 antagonists | 11.1 (3) | 8.2 (42) | 9.8 (6) | 0.486, >0.999 | >0.999, >0.999 |
| Lithium | 0.0 (0) | 0.0 (0) | 0.0 (0) | >0.999, 0.999 | >0.999, >0.999 |
| Carbamazepine | 0.0 (0) | 0.2 (1) | 0.0 (0) | >0.999, >0.999 | >0.999, >0.999 |
| Furosemide | 0.0 (0) | 1.2 (6) | 1.6 (1) | >0.999, >0.999 | >0.999, >0.999 |
| 5-ASA | 3.7 (1) | 0.8 (4) | 0.0 (0) | 0.228, 0.307 | >0.999, >0.999 |
| Rifaximin | 0.0 (0) | 0.0 (0) | 0.0 (0) | >0.999, >0.999 | >0.999, >0.999 |
| Opiates | 0.0 (0) | 0.4 (2) | 0.0 (0) | >0.999, >0.999 | >0.999, >0.999 |
| Anticholinergics | 0.0 (0) | 0.2 (1) | 0.0 (0) | >0.999, >0.999 | >0.999, >0.999 |
| Verapamil | 0.0 (0) | 0.6 (3) | 0.0 (0) | >0.999, >0.999 | >0.999, >0.999 |
| Levothyroxine | 3.7 (1) | 2.9 (15) | 9.8 (6) | 0.566, 0.431 | >0.999, >0.999 |
| Cholestyramine | 0.0 (0) | 0.0 (0) | 0.0 (0) | >0.999, >0.999 | >0.999, >0.999 |
| Chemotherapeutic agents | 0.0 (0) | 0.2 (1) | 1.6 (1) | >0.999, >0.999 | >0.999, >0.999 |
| Monoclonal antinflammatory antibodies | 0.0 (0) | 0.0 (0) | 0.0 (0) | >0.999, >0.999 | >0.999, >0.999 |
| Digoxin | 0.0 (0) | 0.4 (2) | 1.6 (1) | >0.999, >0.999 | >0.999, >0.999 |
| Dopaminergic agents | 0.0 (0) | 0.4 (2) | 0.0 (0) | >0.999, >0.999 | >0.999, >0.999 |
| H2 blockers | 0.0 (0) | 0.0 (0) | 3.3 (2) | >0.999, >0.999 | >0.999, >0.999 |
| Benzodiazepines | 3.7 (1) | 1.4 (7) | 1.6 (1) | 0.339, 0.522 | >0.999, >0.999 |
| Tricyclic antidepressant (TCA) | 3.7 (1) | 0.0 (0) | 1.6 (1) | 0.005, 0.522 | >0.999, >0.999 |
| Antibiotic intake in the last 3 months | 22.2 (6) | 21.4 (119) | 32.8 (20) | >0.999, 0.448 | >0.999, >0.999 |
| Probiotic intake in the last 3 months | 3.7 (1) | 6.1 (31) | 24.6 (15) | >0.999, 0.018 | >0.999, 0.350 |

All results are presented as % (n)

FD, functional dyspepsia; FDR, false discovery rate; GI, gastrointestinal; IBS, irritable bowel syndrome; n, number.

Post-COVID-19 DGBIs groups consists of patients with COVID-19 inflammation who met criteria for DGBIs during follow-up based on Rome IV criteria.

Non-DGBI group consists of patients with COVID-19 inflammation who did not meet DGBI criteria at any point during the follow-up period.

The pre-existing IBS/FD group consists of patients with COVID-19 inflammation and a baseline diagnosis of IBS or FD based on Rome IV criteria.

^1^ P-values for comparisons between post-COVID-19 DGBI and non-DGBI controls.

^2^ P-values for comparisons between post-COVID-19 DGBI and pre-existing IBS/FD patients.
