# Supplementary material for: Long‐Term Impact of COVID‐19 on Disorders of Gut–Brain Interaction: Incidence, Symptom Burden, and Psychological Comorbidities
Source: United European Gastroenterol J. 2025 Mar 21;13(5):798–818. doi: 10.1002/ueg2.70005 (PMC12188378; doi:10.1002/ueg2.70005)
Supplement: Supplementary file 2 — Figure S1 [file UEG2-13-798-s001.pptm]

## Slide 1
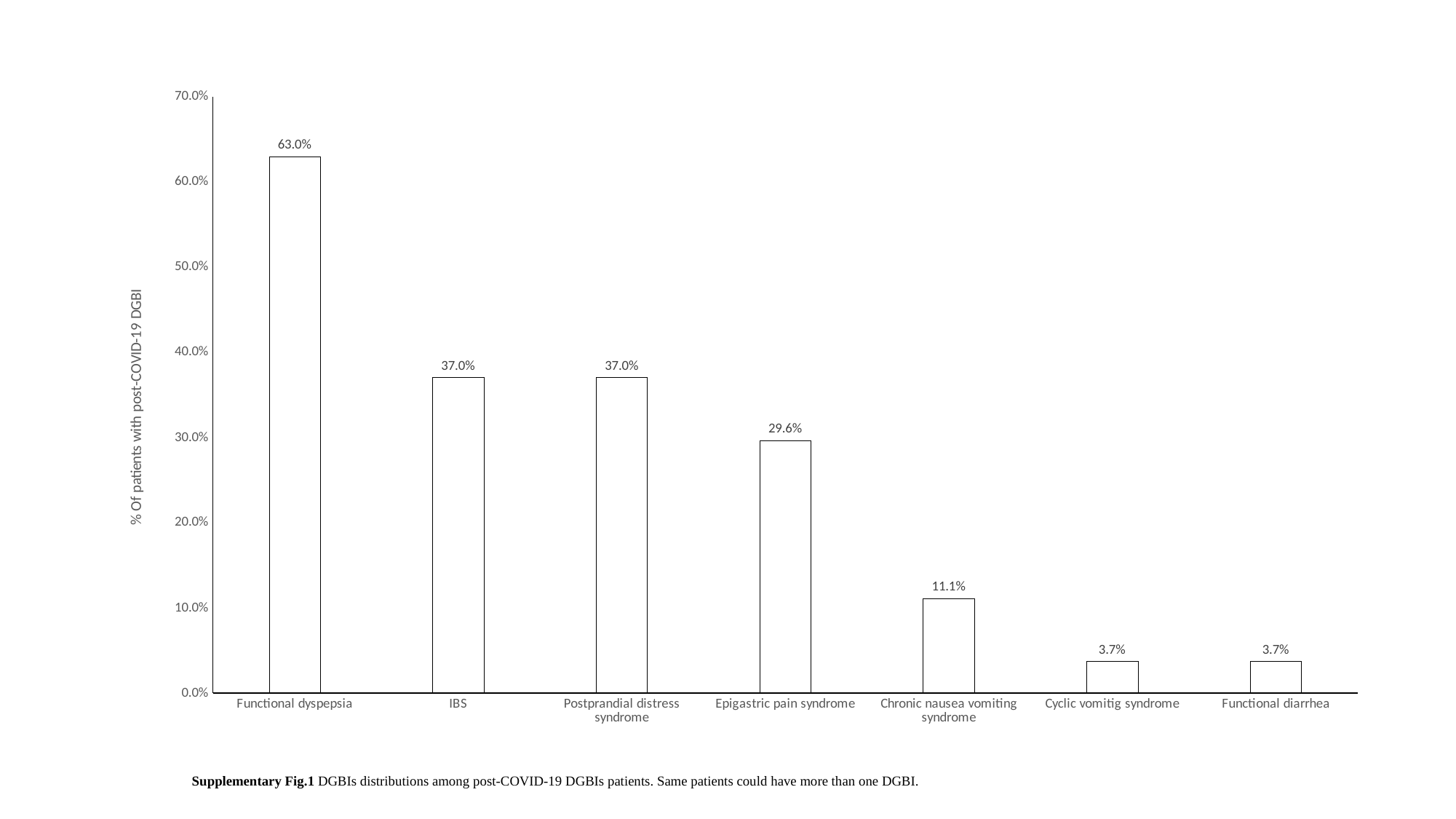

[unsupported chart]
Supplementary Fig.1 DGBIs distributions among post-COVID-19 DGBIs patients. Same patients could have more than one DGBI.
